# Functionalization of an Alginate-Based Material by Oxidation and Reductive Amination

**DOI:** 10.3390/polym13020255

**Published:** 2021-01-14

**Authors:** Ronny G. Huamani-Palomino, Bryan M. Córdova, Elvis Renzo Pichilingue L., Tiago Venâncio, Ana C. Valderrama

**Affiliations:** 1 Laboratorio de Investigación en Biopolímeros y Metalofármacos, Facultad de Ciencias, Escuela Profesional de Química, Universidad Nacional de Ingeniería, Av. Túpac Amaru 210, Lima 15333, Peru; bcordovav@uni.pe; 2 Facultad de Ciencias, Escuela de Química, Universidad Nacional de Ingeniería. Av. Túpac Amaru 210, Lima 15333, Peru; epichilinguel@uni.pe; 3 Laboratório de Ressonância Magnética Nuclear, Departamento de Química, Universidade Federal de Sao Carlos, São Carlos 13565-905, São Paulo, Brazil; venancio@ufscar.br

**Keywords:** alginate, functionalization, oxidation, reductive amination

## Abstract

This research focused on the synthesis of a functional alginate-based material via chemical modification processes with two steps: oxidation and reductive amination. In previous alginate functionalization with a target molecule such as cysteine, the starting material was purified and characterized by UV-Vis, ^1^H-NMR and HSQC. Additionally, the application of FT-IR techniques during each step of alginate functionalization was very useful, since new bands and spiked signals around the pyranose ring (1200–1000 cm^−1^) and anomeric region (1000–750 cm^−1^) region were identified by a second derivative. Additionally, the presence of C_1_-H_1_ of β-D-mannuronic acid residue as well as C_1_-H_1_ of α-L-guluronic acid residue was observed in the FT-IR spectra, including a band at 858 cm^−1^ with characteristics of the N-H moiety from cysteine. The possibility of attaching cysteine molecules to an alginate backbone by oxidation and post-reductive amination processes was confirmed through ^13^C-NMR in solid state; a new peak at 99.2 ppm was observed, owing to a hemiacetal group formed in oxidation alginate. Further, the peak at 31.2 ppm demonstrates the presence of carbon -CH_2_-SH in functionalized alginate—clear evidence that cysteine was successfully attached to the alginate backbone, with 185 μmol of thiol groups per gram polymer estimated in alginate-based material by UV-Visible. Finally, it was observed that guluronic acid residue of alginate are preferentially more affected than mannuronic acid residue in the functionalization.

## 1. Introduction

**Figure 1:**
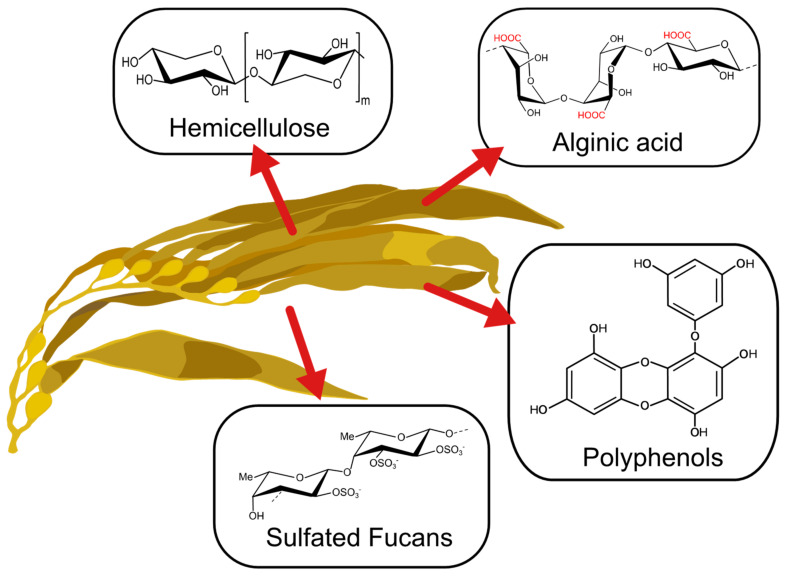
Main compounds present in brown seaweed.

The past decade has seen an increase in the importance of the utilization of algal-based materials and improvement of the physicochemical properties of polysaccharides extracted from the cell wall of brown algae (Figure 1) [1]. This is due to the strong dependence on taxonomic characteristics and the effect of environmental growing conditions on natural sources, which effect its metabolites’ properties [2,3,4,5]. Depending on the type of seaweed, time of harvest, temperature, and other factors, metabolites with novel features can be obtained. These have been used in a wide range of applications [1]. For instance, phlorotannins present in brown seaweeds have been applied as a bioactive agent for their antioxidant, bactericidal and inhibitive properties [6]. Also, the anticoagulant and elicitor properties of fucoidan obtained from brown seaweed have demonstrated promising applications in biomedical fields [7]. Other polysaccharides like alginate have been gaining attention for their application in biomedical and environmental fields [8,9]. 

Alginate is a linear anionic copolymer composed of [1,2,3,4] β-D-mannuronic’ acid (M) and α-l-guluronic acid (G). This polysaccharide is mainly extracted from brown seaweeds and is characterized by its random block distribution, which offers unique properties depending on the length of the polymeric chain and the presence of different stretches of alternating or homogeneous M and G sequences, referred to as MG-blocks, MM-blocks or GG-blocks. The M/G ratio is an important parameter that gives crucial information associated with the block composition of alginates from a particular brown seaweed. It must be mentioned that the block distribution depends exclusively on the species, habitat, season of harvest and salinity of water [10,11]. Thus, it is essential to refer to the algae’s background for chemical modification purposes.

Alginates are employed industrially for their viscosifying properties, water binding capacities and gelling properties [12], which correlate with the amount of GG blocks in the polymer chain as well as the presence of divalent cations in the aqueous medium [13]. These properties have allowed us to widen alginate applications, especially in biomedical areas, including wound healing [14,15,16], cell microencapsulation [17,18,19] and drug delivery systems [20,21,22]. However, the main limitation regarding these applications lies especially on low biocompatibility and poor mechanical properties, preventing the utilization of unmodified alginates for sophisticated biomedical areas such as cell immobilization [23,24]. Taking into account these drawbacks, functionalization of alginate-based materials emerges as an excellent option to enhance the quality and long-term stability of alginates without restricting the action of some specific properties characteristic of them [25]. Furthermore, the physical properties of alginate-based materials can be improved while avoiding the incorporation of other materials, such as carbon nanomaterials [26,27] or nanocellulose [28]. Different studies have reported that chelating [29], permeation [30] and, especially, mucoadhesive [31,32] properties can be improved when sulphur-containing molecules are attached to the alginate backbone. In particular, the synthesis of thiol-containing biopolymers offers numerous attractive features for a variety of biomedical applications in which the biomaterial can perform as a drug delivery system and bioadhesive [33,34].

In this regard, Bernkop-Schnürch et al. synthetized thiomers from the implantation of thiol groups in different polysaccharides, such as chitosan, pectin and alginate [23,35,36]. The main pathway to obtain thiomers in alginate is to render the carboxylic acid group into an amine-reactive reagent via carbodiimide coupling [31,37], but the main drawback is that this procedure tends to generate side products during the functionalization process [37,38]. On the other hand, other chemical routes that involve oxidation and reductive amination processes [39,40] have shown promising properties, including increasing the degradability and chain flexibility of the polymer [41,42]. Novel materials obtained have demonstrated their versatility in varied fields, but most of these are not focused on structural changes upon oxidation and subsequent grafting. Based on this, the present study aims to provide an efficient method to functionalize the alginate in two steps via oxidation and post reductive amination reaction for evaluating the preference of uronic residue involved in this process.

## 2. Materials and Methods

### 2.1. Materials

Commercial-grade sodium alginate (Mw = 124.2, Mn = 57.8 and PI = 2.1 (Table S1) obtained by SEC-MALS (Figure S1)) and L-cysteine hydrochloride (HSCH_2_CH(NH_2_)COOH∙HCl) were obtained from Sigma Aldrich Chemistry (St. Louis, MO, USA). All other reagents were supplied by Merck (KGaA, Darmstadt, Germany) except for ethylene glycol (98%, Fermont).

### 2.2. Purification of Commercial Alginate

The alginate (Alg) was purified in order to remove polyphenol compounds that are usually present in commercial-grade products. For this purpose, a solution of 1% was treated with n-butanol in a ratio of 3 to 2. This mixture was sonicated for 1 h, and then the solution was reposed until the formation of 2 phases. Finally, the aqueous phase was separated by decantation and lyophilized for 24 h to obtain the purified alginate (AlgP).

### 2.3. Functionalization of Purified Alginate with Cysteine

**Figure 2:**
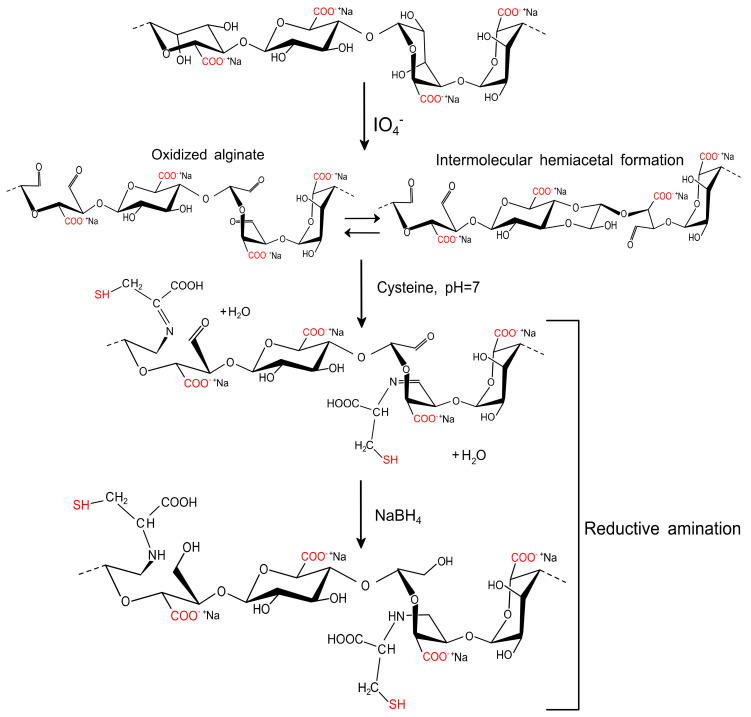
Proposed reaction for the functionalization of sodium alginate with cysteine employed by oxidation and reductive amination.

The functionalization of AlgP with cysteine was prepared following the procedure reported by our group previously, but with several modifications [32]. First, 4 g of AlgP was dissolved thoroughly in 170 mL of distilled water prior the addition of 0.2 mol L^−1^ NaIO_4_ (30 mL). This solution was stirred for 6 h in complete darkness to prevent undesired reactions [43]. Then, 1 mL of ethylene glycol was added and stirred for 30 min under the same conditions. The solution containing the oxidized alginate was dialyzed against deionized water by using a cellulose membrane (cut-off molecular weight of 12 kDa) until the conductivity of the aqueous medium was less than 10 μS cm^−1^. Next, the oxidized alginate (AlgPO) was obtained by freeze-drying for 36 h. Afterward, 40 mL of 0.1 mol L^−1^ phosphate buffer solution (PBS, pH 7.4) was used as the medium to dissolve 0.4 g of AlgPO before the addition of 0.2 mol L^−1^ cysteine solution (10 mL). Finally, the mixture was stirred for 24 h at room temperature prior to the addition of 0.05 mol L^−1^ NaBH_4_ (10 mL). The solution reacted for 12 h and a functionalized alginate-based material (AlgPOS) was obtained through precipitation in ethanol and a freeze-drying process (Figure 2).

## 3. Characterization

### 3.1. Evaluation of Polyphenols by UV-Vis

For this analysis, both phases (organic and aqueous) were collected and analyzed by UV-Vis in the range of 500–200 nm. UV-Vis analyses were performed with a UV-1800 Shimadzu scanning spectrophotometer.

### 3.2. Evaluation of Thiols by UV-Vis

The presence of thiol groups was quantified spectrophotometrically using Ellman’s reagent (DTNB, 5,5-dithio-bis (2-nitrobenzoic acid)) [44]. Then, 40.2 mg of AlgPOS was dissolved in a stock solution of 0.1 mmol L^−1^ DTNB prepared in PBS buffer. The quantity of thiol groups was estimated from a standard curve of L-cysteine.

### 3.3. FT-IR Measurements

Fourier-Transform Infrared (FT-IR) measurements were performed during each step of alginate functionalization (AlgP, AlgPO and AlgPOS) using a Shimadzu IR Prestige-21 spectrometer with Attenuated Total Reflection (ATR). The spectra were acquired (64 scans/sample) in the range of 4000–600 cm^−1^ at room temperature with a resolution of 4 cm^−1^. Derivations, including a Savitzky–Golay algorithm with 23 smoothing points, were analyzed with OriginLab 9.0 software.

### 3.4. ^1^H NMR and HSQC Analyses of AlgP

In order to reach a degree of polymerization around 10–30, AlgP was hydrolyzed according to the procedure reported in the literature [45]. Then, 10 mg of hydrolyzed AlgP wwas dissolved in 500 μL of D_2_O. Next, TMSP-d_4_ -3-(Trimethylsilyl)-propionic-2,2,3,3-d_4 -_acid sodium salt was added as an internal standard for the chemical shift. The ^1^H NMR spectrum was recorded on a Bruker Avance III-400 spectrometer at 80 °C and processed with TopSpin 3.2 software (Bruker BioSpin, Billerica, MA, USA).

### 3.5. Analysis of Alginate Derivatives by Solid State ^13^C NMR

The analyses by solid state ^13^C NMR were performed in a Bruker Avance III-400 operating at 9.4 T magnetic field, ν (^1^ H) = 400 MHz. The ^13^C resonance frequency was 100.57 MHz with a pulse sequence (cross-polarization on magic-angle spinning (CP-MAS)) and CP-MAS with total sideband suppression (CP-MAS-TOSS). A high-power decoupling field was set at 83.3 kHz [P_90_ (^1^H) = 3 µs], and adamantane (38.5 p pm for CH_2_ resonance) was used as an external reference for adjustment of the ^13^C chemical shift.

## 4. Results and Discussion

### 4.1. Analysis of the Starting Material

**Figure 3:**
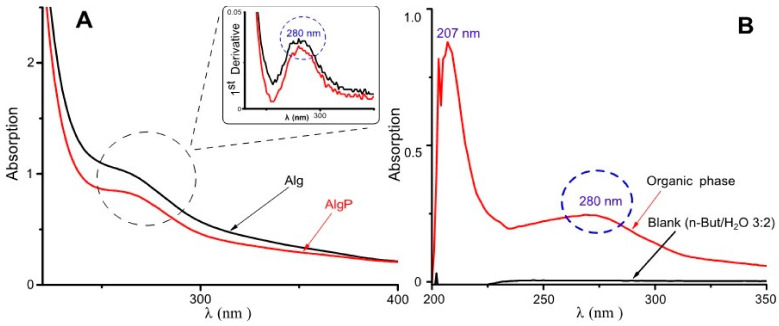
UV–Visible spectra for alginate purification. (**A**) Aqueous phase with the first derivative. (**B**) Organic phase.

Purification of commercial-grade alginates ought to be carried out prior to alginate functionalization, since production of this polysaccharide is based exclusively on extraction from brown seaweeds [46]. In this regard, short aliphatic chains like tert-butanol have been employed in the three-phase partitioning method for the bioseparation of proteins [47]. In contrast, n-butanol can be used for separating low-molecular substances present in alginates, including aromatic acids, hydroxyl acids, carbohydrates and polyols [48]. Hence, in this study, n-butanol was used, aiming to remove a significant quantity of substances commonly found in commercial-grade alginates. In Figure 3A, at around 280 nm, a signal can be seen probably by π–π* transitions attributed to the ring benzene present in polyphenols [6,49]. This asseveration is evidenced in the spectrum by its first derivative. Moreover, in Figure 3B, the organic phase shows its capability of extracting low-molecular-weight molecules and polyphenols owing to the presence of bands around 207 nm and 280 nm, attributed to the ring benzene present in phlorotannins [50,51]. Consequently, these bands could be assigned to polyphenols or similar molecules characterized by having a ring benzene, which may still remain in alginates.

**Figure 4:**
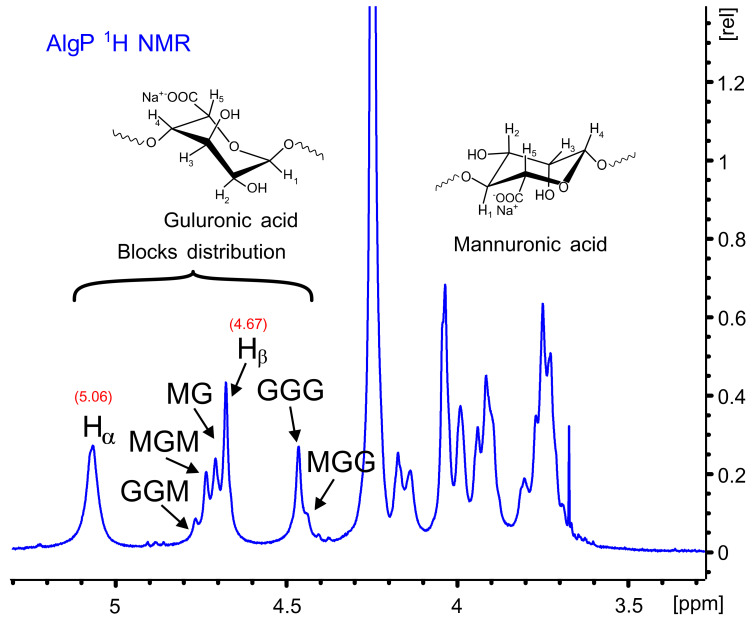
^1^H NMR spectrum of AlgP.

Figure 4 shows the ^1^H NMR spectrum of AlgP for evaluating the M/G ratio according to the method described by S. Pawar and K. Edgar [52]. This parameter was calculated from the signals in the anomeric region (4.4–5.5 ppm) through the relationship of G and M block distribution [53,54]. In this case, the M/G value of commercial sodium alginate after the purification process with n-butanol is 1.02 (Table S2). This parameter directly influences the capacity to form gels through crosslinking reactions with divalent ions, since it is a well-known fact that alginates with a low M/G ratio produce stronger structures due to the high affinity of G blocks towards calcium ions. On the contrary, alginates with a high M/G ratio possess promising elastic properties and are able to form acidic gels [55,56]. Considering that AlgP is composed of almost 50% of each uronic acid, oxidation and reductive amination processes could be studied to evaluate the susceptibility of hemiacetal formation in G or M blocks, as well as their effect on cysteine after the reductive amination process. The application of solid state NMR spectroscopy for analyzing the linking of nitrogen-containing molecules and the presence of thiol groups have been reported elsewhere [57]. For this reason, ^13^C CP-MAS NMR spectra of alginate derivatives were studied in detail and discussed in terms of the results obtained by second derivative FT-IR analysis.

**Figure 5:**
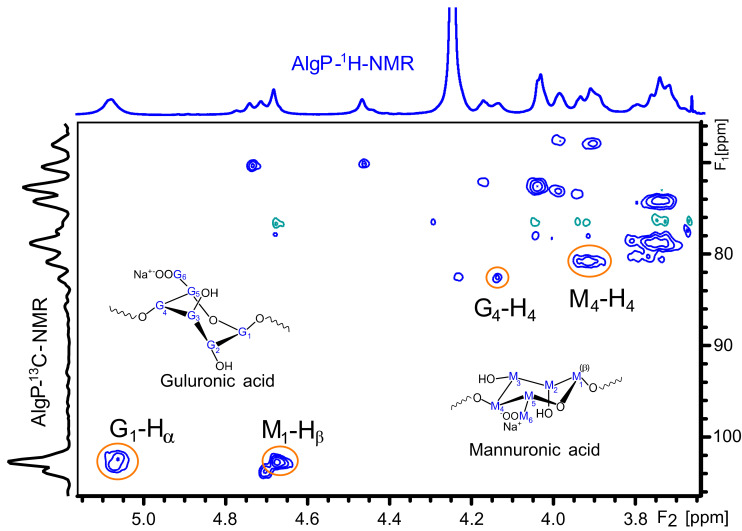
HSQC spectrum of purified alginate (AlgP).

The ^1^H and ^13^C NMR chemical shifts of AlgP are depicted in Table 1. For a better interpretation of AlgP’s structure, Figure 5 displays the ^13^C/^1^H HSQC spectrum of AlgP in order to evaluate the correlation between ^13^C-^1^H (Table 1). As can be observed, the 103.92/4.67 ppm correlation was assigned to C_1_/H_1_ of β-D-mannuronic acid residues, whereas the signal at 102.70/5.06 ppm was attributed to C_1_/H_1_ of α-L-guluronic acid residue. The 80.78/3.91 ppm and 82.63/4.13 ppm correlations were assigned to C_4_/H_4_ of manuronic (AlgP^a^) and guluronic (AlgP^b^) acid residues in AlgP.

### 4.2. Evaluation of Alginate Functionalization

#### 4.2.1. FT-IR spectroscopy

**Figure 6:**
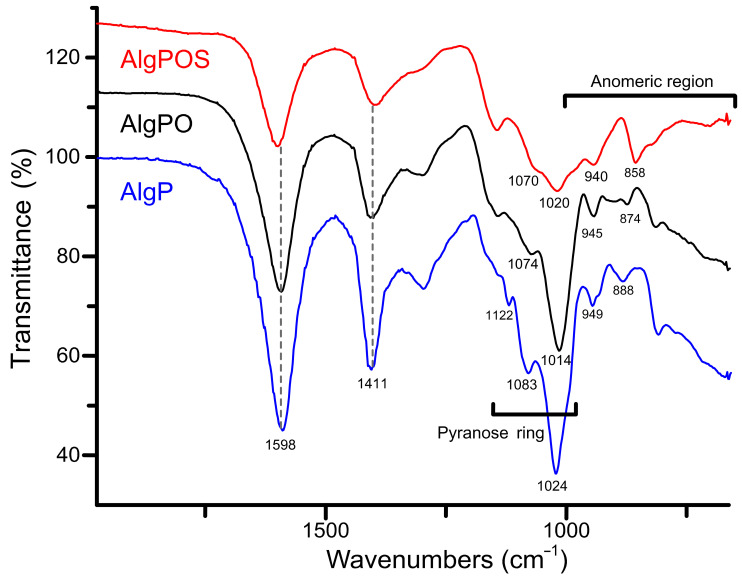
ATR-IR spectra of the starting material (AlgP), oxidized alginate (AlgPO) and alginate-based material (AlgPOS).

Figure 6 depicts three different ATR-IR spectra obtained during each step of alginate functionalization. The typical signals around 1598 cm^−1^ and 1411 cm^−1^ were assigned to asymmetric and symmetric movements of carboxylate groups present in the structure despite the functionalization process. According to this, carboxylate groups are not affected considerably by oxidation and reductive amination processes. In contrast, the band associated with C-O stretching vibration of pyranose rings was clearly affected, as is demonstrated with the chemical shifts from 1024 cm^−1^ to 1014 cm^−1^ (after periodate oxidation), and then to 1020 cm^−1^ due to the structural modification with cysteine. The bands at 1122 cm^−1^ and 1083 cm^−1^ assigned to C-O and C-C stretching vibrations of pyranose rings [58] were also affected by structural changes produced by functionalization with cysteine.

With regard to the anomeric region around 1000–750 cm^−1^, the spectra exhibit a band at 949 cm^−1^ assigned to C-O stretching vibration of uronic acid residues, while the band at 888 cm^−1^ is assigned to C_1_-H deformation vibration of β - D mannuronic acid residues [59].

**Figure 7:**
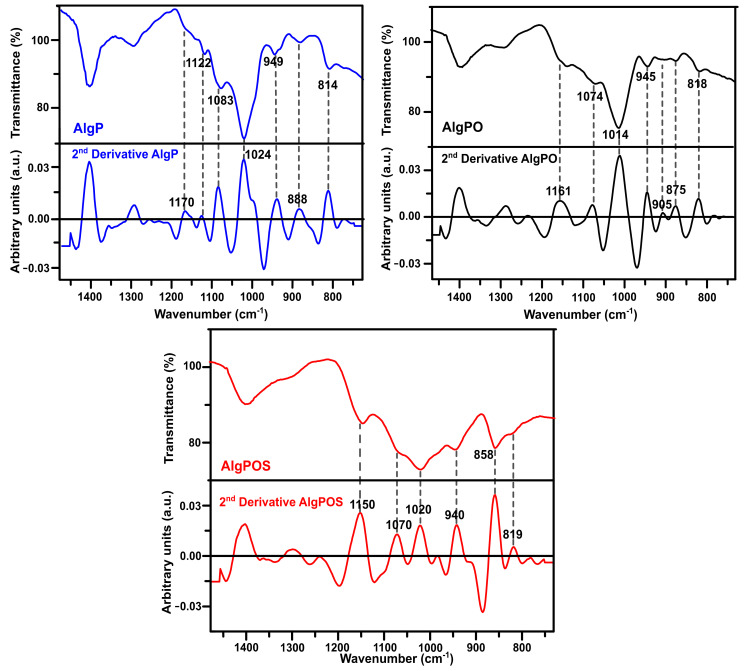
Second-derivative ATR-IR spectra of the starting material (AlgP), oxidized alginate (AlgPO) and alginate-based material (AlgPOS).

In order to improve the resolution of overlapping bands in the normal FT-IR spectrum [60], the second-derivative FT-IR technique was applied for analyzing alginate derivatives—as this method has been widely used for structural analysis of macromolecules [32,59,61]. Thus, in Figure 6, the C-O and C-C stretching vibrations were found at 1122 cm^−1^ for AlgP. This band was considerably affected by oxidation, as well as the reductive amination process, and, therefore, this band vanished in AlgPO and AlgPOS spectra. Another similar situation is observed in Figure 7 since the band at 1170 cm^−1^ (second derivative) is assigned to the C-O stretching vibration of the glycosidic linkage of AlgP; this band was affected in each step of the functionalization, resulting in a chemical shift towards 1161 cm^−1^ for AlgPO (second derivative), whereas this band was at 1150 cm^−1^ for AlgPOS (second derivative). The C-O and C-C stretching vibrations of pyranose rings (observed at 1083 cm^−1^) were shifted to 1074 cm^−1^ (second derivative) and 1070 cm^−1^ (second derivative) because of oxidation and the reductive amination process, respectively. These bands are possibly shifting due to the Malaprade reaction, as periodates do indeed cleave the pyranose ring between C_2_ and C_3_. This procedure is the best route to oxidize diols into aldehydes under dark conditions (Figure 3). Additionally, the reductive amination process using cysteine leads to the formation of imines, which are reduced with sodium borohydride (Figure 3). It must be noted that the second-derivative technique is very useful for studying the anomeric region, where bands at 949 cm^−1^ (AlgP), 945 cm^−1^ (AlgPO) and 940 cm^−1^ (AlgPOS, second derivative), associated with C-O stretching vibration, shifted to lower vibration frequencies as a consequence of the new environment caused by the ring opening and post introduction of cysteine molecules into the polymer chain.

According to Matsuhiro et al. [62], the alginate always exhibits two characteristic bands associated with the C_1_-H anomeric of β-D-mannuronic acid at around 888 cm^−1^, and another band at 902 cm^−1^ characteristic of C_1_-H of α-L-guluronic acid residue. In AlgPO and AlgPOS, it could be observed how these signals were affected due to the degradation process of blockchains caused by the functionalization process (Figure 7) [63]. Furthermore, the band at around 814 cm^−1^ is assigned to COH, CCH and OCH stretching vibrations of α-L-guluronic acid residues, with a contribution of bending deformation vibrations of C-O-C glycosidic linkages in homopolymeric blocks [58,59]. This band is clearly observed at 818 cm^−1^ in the normal ATR-IR spectrum of AlgPO. However, without the utilization of the second-derivative technique, it would be challenging to detect the signal at 819 cm^−1^, which was overlapped in the classical ATR-IR spectrum of AlgPOS. Finally, the band observed at 858 cm^−1^ may be assigned to N-H stretching vibration as a consequence of cysteine molecules [64]. The presence of thiol groups in the alginate structure was corroborated through the Ellman method (Figure S2), reporting 185 μmol thiol groups per gram of AlgPOS, as shown in Table S3.

#### 4.2.2. Analysis of Alginate and Its Derivatives by ^13^C NMR in Solid State

**Figure 8:**
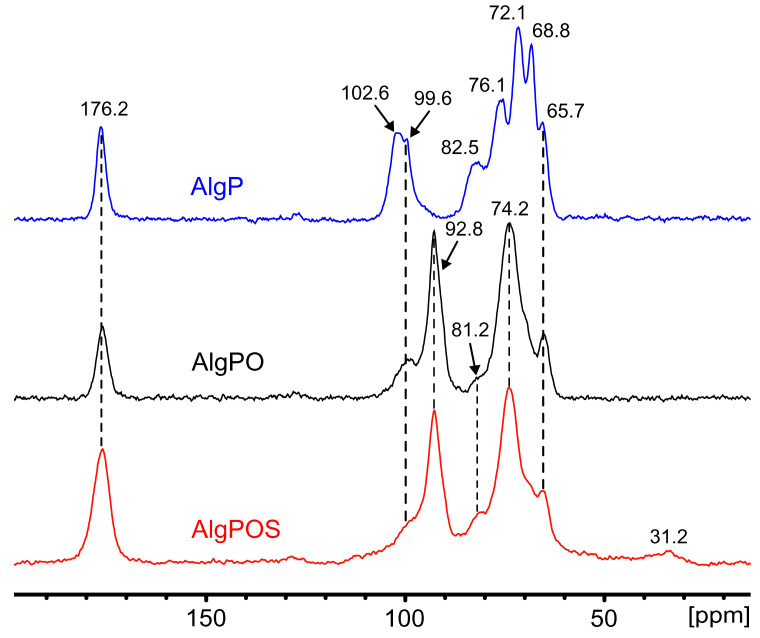
^13^C NMR in solid state in each step of functionalization. i. Starting material (AlgP), ii. Oxidized alginate (AlgPO) and iii. Alginate after reductive amination (AlgPOS).

The effect ascribed to the chemical reactions during alginate functionalization was studied by the FT-IR technique and confirmed by using ^13^C NMR in solid state. Figure 8 displays the spectrum of AlgP, and its characteristic peaks are assigned in Table 2. Owing to the oxidation produced by the utilization of sodium metaperiodate as an oxidizing agent, a new peak emerges at 92.8 ppm attributed to hemiacetal groups formed by intramolecular interactions between aldehyde groups of oxidized residues and hydroxyl groups of unoxidized hydroxyl moieties that were situated in neighboring positions of the same polymer chain [65]. To achieve an effective functionalization via reductive amination, crucial factors must be controlled to form the imine bond successfully, e.g., pH, reaction time and the amount of nitrogen-containing molecules [32]. As we demonstrated in a previous work, thiosemicarbazide (NH_2_-NH-(C=S)-NH_2_) promotes crosslinking reactions between both terminal primary amine groups present in the molecule and oxidized moieties of alginate [66]. Thus, a strict control of pH ≈ 7 and reaction time above 24 h can guarantee the formation of imine bonds between cysteine molecules and hemiacetal groups, which revert back to aldehyde form in aqueous medium during the reductive amination process [32]. In addition, a high concentration of buffer makes it feasible to avoid the formation of thiazolidine, a side product that is produced by the interaction between thiols and aldehydes [67]. In these conditions, this side reaction would not be favorable on thermodynamic grounds [32,67]. On the other hand, it has been previously reported that the -CH_2_-SH moiety appears at 28.8 ppm [57]. Thus, we have considered that the peak at 31.2 ppm is attributed to -CH_2_-SH of the cysteine attached to the alginate backbone. This new peak is present in the spectra due to the efficient reductive amination process, performed in order to achieve the functionalization of alginate. The resonances of both the M-6 and G-6 contribution at 176.2 ppm assigned to carboxylate groups have not been altered, which is clear evidence that sodium metaperiodate is a suitable oxidizing agent to convert diols into aldehydes without affecting carboxylate groups. The pyranose region showed considerable structure changes associated with guluronic residue. The signals corresponding to G-4 grew smaller along each step of the functionalization process. Several signals obtained by NMR in solid state consisted of contributions from more carbon sites within each mannuronic and guluronic acid residue, due to chemical shift distribution [53]. Thus, the signal of 68.8 ppm in AlgP contains a contribution of G-3 and G-5, which is noticeably affected by periodate oxidation in AlgPO and AlgPOS (Table 2). Additionally, the intensity of the peaks at 102.6 ppm (G-1) and 99.6 ppm (M-1), associated with the C_1_-H anomeric of α-L-guluronic acid residue and C_1_-H of β-D-mannuronic acid, respectively [53], have both shown a decrease due to rupture of glycosidic linkage, due to severely affected carbon atoms associated with α-L-guluronic acid residues as displayed in Figure 8.

Conversely, the signals corresponding to M have not shown a significant change, as the peaks at 74.2 ppm could be a contribution of both signals at 72.1 ppm and 76.1 ppm, assigned to M-2 and M-4, respectively. These results suggest that G units of AlgPO are preferentially oxidized since G units were greatly affected rather than M—in spite of being present in the alginate structure in almost equal proportions (M/G ratio 1.02). Nevertheless, other factors like block distribution might be more important than the M/G ratio, especially when the M/G ratio gives an almost equal proportion of M and G blocks, as is reported in this work. Since G residue is more reactive than M to cleavage, the stability of these oxidized groups will depend on their neighbors. These might stabilize them, forming hemiacetal groups, or might be affected by a fast hydrolyzation, which would lead to a decrease in alginate-based material formation [68,69]. To conclude, the effect of the reducing agent during the reductive amination process depends on several factors [70] and, thus, the utilization of a specific borohydride derivative ought to be explored, taking into account that sodium cyanoborohydride generates the presence of HCN, NaCN and cyanoborohydride derivatives that are highly toxic for the environment and human beings [71,72]. Hence, according to the literature, sodium borohydride is a low-cost and efficient reagent that can be used during the reductive amination process under different conditions with good yields [73,74]. 

## 5. Conclusions

In summation, the functionalization of purified sodium alginate was achieved successfully via oxidation and the reductive amination process. In order to evaluate the chemical modification using a purified raw material (AlgP), polyphenols and phlorotannins were removed from commercial-grade alginate using n-butanol. Characterization of AlgP shows a M/G of 1.02, whereas HSQC studied correlations of ^1^H-^13^C. AlgPOS demonstrated cysteine covalently bound to the alginate with a coupling around 185 μmol of attached thiol groups per gram of polymer as estimated by UV-Vis. FT-IR and solid state ^13^C NMR analyses confirmed the functionalization of alginate, as peaks were significantly displaced from their natural position. Around the pyranose and fingerprint regions, we observed two peaks at 99.2 ppm and 31.2 ppm, respectively. These peaks were attributed to hemiacetal formation due to the interaction of activated/deactivated aldehydes obtained by oxidation, whereas the attaching of cysteine to the alginate backbone caused an upfield resonance characteristic of CH_2_-SH moieties from cysteine. The chemical shifts and vanished signals to G2 and G4 and G1, G3 and G5, respectively, in AlgPOS by ^13^C NMR demonstrated the susceptibility of guluronic groups in the process of functionalization.

## Figures and Tables

**Table 1:** Assignment of the chemical shifts of AlgP ^1^H NMR.

Sample	δ (ppm)										
	H-1	C-1	H-2	C-2	H-3	C-3	H-4	C-4	H-5	C-5	C-6
AlgP ^a^	4.67	103.92	4.02	72.58	3.74	74.11	3.91	80.78	3.75	78.79	175.4
AlgP ^b^	5.06	102.67	3.98	67.78	4.17	72.23	4.13	82.63	4.46	70.14	176.1

^a^ Chemical shifts for mannuronic acid residue in AlgP. ^b^ Chemical shifts for guluronic acid residue in AlgP.

**Table 2:** Assignment of the chemical shifts by ^13^C NMR in solid state.

	δ (ppm)													
	Anomeric		Pyranose								Carboxylate		Hemiacetal	-CH_2_-SH
Assignment of M (mannuronic) or G (guluronic) carbons	G-1	M-1	G-4	M-5	M-4	M-2	M-3	G-3	G-5	G-2	M-6	G-6		
**AlgP**	102.6	99.6	82.5	76.1		72.1		68.8		65.7	176.2		-	-
**AlgPO**	-	99.6	81.2	74.2				-		65.7	176.2		92.8	-
**AlgPOS**	-	99.6	81.2	74.2				-		65.7	176.2		92.8	31.2

## Data Availability

The data presented in this study are available on request from the corresponding author.

## Supplementary Materials

The following are available online at https://www.mdpi.com/2073-4360/13/2/255/s1, Figure S1: Refractive index chromatogram of commercial sodium alginate used as starting material., Figure S2. UV-Vis spectra of cysteine standards (E1, E2 and E3) and AlgPOS. Evaluation of the thiol group by Ellman’s reaction. Table S1. Values obtained of commercial sodium alginate by SEC-MALS., Table S2. Values obtained of commercial sodium alginate by ^1^H NMR., Table S3. Values obtained of AlgPOS by UV-Vis.
