# Supplementary material for: Functionalization of an Alginate-Based Material by Oxidation and Reductive Amination
Source: Polymers (Basel). 2021 Jan 14;13(2):255. doi: 10.3390/polym13020255 (PMC7828833; doi:10.3390/polym13020255)
Supplement: Supplementary file 1 [file polymers-13-00255-s001.pdf]

Supporting information

Functionalization of an alginate-based Material by Oxidation and Reductive Amination

Ronny G. Huamani-Palomino[1,*], Bryan M. Córdova[1], E. Renzo Pichilingue L.[2], Tiago Venâncio[3], and Ana C. Valderrama[1,*]

[1] Laboratory Investigation of Biopolymers and Metallopharmaceuticals (LIBIMET), Faculty of Sciences, Chemistry Department, National University of Engineering. Av. Túpac Amaru 210, 15333, Lima 25, Peru; bcordovav@uni.pe
[2] National University of Engineering Av. Túpac Amaru 210, 15333, Lima 25, Peru; epichilinguel@uni.pe
[3] Laboratório de Ressonância Magnética Nuclear, Departamento de Química, Universidade Federal de Sao Carlos, São Carlos, São Paulo CP 676, 13565-905, Brazil; venancio@ufscar.br
* Correspondence: rohuamanip@uni.pe (R.G.H.); ana.valderrama.n@uni.edu.pe (A.C.V.)

The molecular weight of commercial sodium alginate was evaluated by Size-exclusion chromatography (SEC) with online multi-angle static laser light scattering (MALLS), which were performed at ambient temperature on an HPLC system consisting of a solvent reservoir, on-line degasser, HPLC isocratic pump, automatic sample injector, pre-column, and a G6000PW main column. The column outlet was connected to a Dawn HELEOS-II multiangle laser light scattering photometer (Wyatt, U.S.A.) ($\lambda 0$ = 663.8 nm) followed by Shodex RI-501 refractive index detector. The eluent was 0.15 mol L^{-1} $NaNO_3$, 0.01 mol L^{-1} EDTA (pH = 6.0) and the flow rate was 0.5 mL min^{-1}. Samples (1 mg mL^{-1}) were filtered (pore size 0.45 μm) before injection and each sample were analyzed twice with injection volume 50 and 100 μL. Data were collected and processed (with dn/dc = 0.150 mL g^{-1}) using the Astra (v. 7.3.0) software (Wyatt, U.S.A.). The analysis was performed according to the ASTM protocol nr. F2602 – 13.

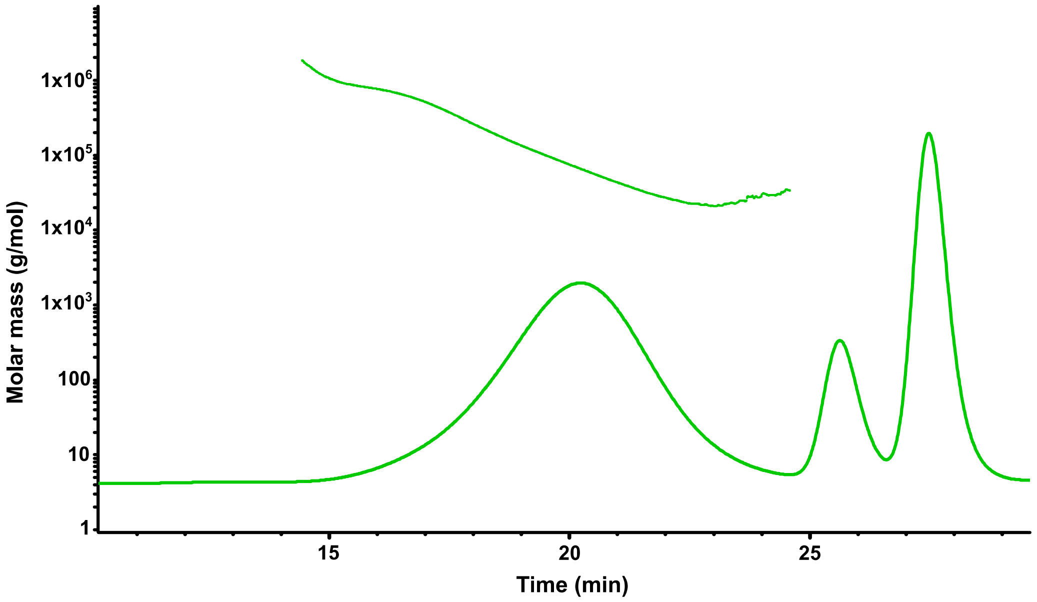

Figure S1. Refractive index chromatogram of commercial sodium alginate used as starting material.

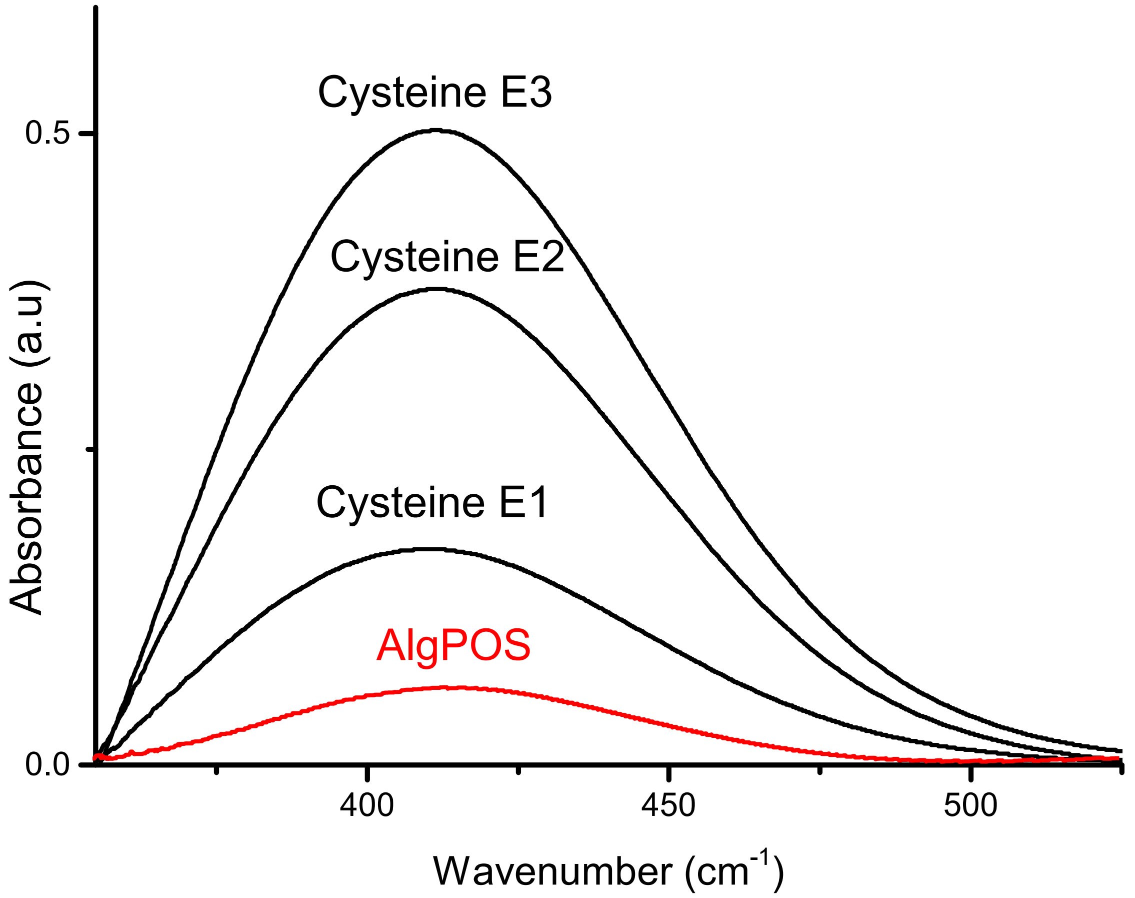

Figure S2. UV-Vis spectra of cysteine standards (E1, E2 and E3) and AlgPOS. Evaluation of thiols group by Ellman's reaction.

Table S1. Values obtained of commercial sodium alginate by SEC-MALS

Sample	Mn	Mw	PI (Mw/Mn)
Sodium alginate (Sigma Aldrich)	58.6	124.7	2.1
	57.0	123.7	2.2
**Average**	**57.8**	**124.2**	**2.1**

Table S2. Values obtained of commercial sodium alginate by ^{1}H NMR

Sample	F_G	F_M	F_{GG}	F_{GM}	F_{MM}	F_{GGM}	F_{MGM}	F_{GGG}	N(G>1)	M/G
Sodium alginate (Sigma Aldrich)	0.49	0.50	0.30	0.18	0.31	0.07	0.11	0.24	5.33	1.02

Table S3. Values obtained of AlgPOS by UV-Visible.

Sample	Absorbance	[C] (μM)	V(mL)
Cysteine E1	0.171	24.6	10
Cysteine E2	0.377	49.2	10
Cysteine E3	0.503	61.5	10
AlgPOS	0.086	74.7	200
